# *Carthamus tinctorius* Enhances the Antitumor Activity of Dendritic Cell Vaccines via Polarization toward Th1 Cytokines and Increase of Cytotoxic T Lymphocytes

**DOI:** 10.1093/ecam/nen068

**Published:** 2011-01-03

**Authors:** Jia-Ming Chang, Le-Mei Hung, Yau-Jan Chyan, Chun-Ming Cheng, Rey-Yuh Wu

**Affiliations:** Division of Research and Development, Development Center for Biotechnology, Xizhi City, Taipei County, Taiwan 221, R.O.C., Taiwan

## Abstract

*Carthamus tinctorius* (CT), also named safflower, is a traditional Chinese medicine widely used to improve blood circulation. CT also has been studied for its antitumor activity in certain cancers. To investigate the effects of CT on the dendritic cell (DC)-based vaccine in cancer treatment, cytokine secretion of mouse splenic T lymphocytes and the maturation of DCs in response to CT were analyzed. To assess the antitumor activity of CT extract on mouse CD117^+^ (c-kit)-derived DCs pulsed with JC mammal tumor antigens, the JC tumor was challenged by the CT-treated DC vaccine *in vivo*. CT stimulated IFN-**γ** and IL-10 secretion of splenic T lymphocytes and enhanced the maturation of DCs by enhancing immunological molecule expression. When DC vaccine was pulsed with tumor antigens along with CT extract, the levels of TNF-**α** and IL-1**β** were dramatically increased with a dose-dependent response and more immunologic and co-stimulatory molecules were expressed on the DC surface. In addition, CT-treated tumor lysate-pulsed DC vaccine reduced the tumor weight in tumor-bearing mice by 15.3% more than tumor lysate-pulsed DC vaccine without CT treatment. CT polarized cytokine secretion toward the Th1 pathway and also increased the population of cytotoxic T lymphocytes *ex vivo*. In conclusion, CT activates DCs might promote the recognition of antigens and facilitate antigen presentation to Th1 immune responses.

## 1. Introduction

*Carthamus tinctorius* (CT), also named safflower, is a traditional Chinese medicine widely used to improve blood circulation [1], extending the coagulation time in mice and exhibiting a significant antithrombotic effect [2]. However, CT is used not only for its traditional medicinal purposes but is also effective for treating breast cancer [3]. The oil extracted from the seed of CT is reported to contain alkane-6,8-diols, which have the activity to inhibit 12-*O*-tetradecanoylphorbol-13-acetate-induced tumor promotion in two-stage carcinogenesis in mouse skin. In addition, *N*-feruloylserotonin and *N*-(*p*-coumaroyl) serotonin strongly inhibit the melanin production of *Streptomyces bikiniensis* and B16 melanoma cells [4, 5]. These compounds are suggested to have potential antitumor effects. In addition, CT also is neuroprotective for cerebral ischemic injury *in vivo* and *in vitro* [2]. Recently, *N*-(*p*-coumaroyl)serotonin and *N*-(*p*-coumaroyl)tryptamine, active ingredients in CT, were shown to strongly inhibit the production of proinflammatory cytokines (IL-1*α*, IL-1*β*, IL-6, IL-8 and TNF-*α*) from lipopolysaccharide-stimulated human monocytes [6]. Notably, extracts of CT exhibit a vast diversity of bioactivities, including immunomodulation, anti-infarction, antiallergic, anti-inflammatory and antiestrogenic effects, as well as functioning as a hemostatic agent to promote blood coagulation.  Table 1 summarizes the diverse bioactivities of CT and delineates the therapeutic implications for this potent herbal medicine.

Dendritic cells (DCs) are professional antigen-presenting cells that stimulate immune responses by presenting endogenous or exogenous antigens to T-helper lymphocytes [31]. Immature DCs can be differentiated from monocytes and bone marrow progenitor cells by treatment with GM-CSF and IL-4 [32, 33], after which the cells gain strong phagocytic activity but not antigen presentation activity. Immature DCs are stimulated with maturation signals to express more surface immunological molecules (such as CD86, CD80, MHC-I and MHC-II) for antigen presentation, leading to a strong immune response against foreign antigens [34]. TNF-*α* and CD40L can stimulate immature DCs to mature *in vitro* and may show potential for clinical use [35], suggesting that enhancement of DC maturation is required for improvement of DC immunotherapy. In the clinic, DC-based immunotherapies are being intensively studied for their application in the treatment of certain diseases, including cancer and infectious diseases [36–38]. Scientists are trying to improve the phagocytic activity or antigen presentation activity of DCs for use in immunotherapy [39, 40]. Certain herbal medicines can also improve DC function [41]. To date, CT has been used as a folk medicine in cancer adjuvant therapy, but its function has not been proven. In this study, we studied the immunomodulatory effects of CT on cytokine secretion and surface immunologic molecules of DCs *in vitro* and antitumor activities of CT-treated DCs in an animal model of breast cancer.

## 2. Materials and Methods

### 2.1. Cell Cultures and Reagents

JC cells (mouse mammary adenocarcinoma) were purchased form American Type Culture Collection (ATCC, USA) and cultured in MEM medium (Gibco, Invitrogen Inc., USA) supplemented with 10% fetal bovine serum (FBS) (Hyclone, USA). GM-CSF and IL-4 were purchased from the PeproTech Inc. (NJ, USA). Phycoerythrin (PE)-hamster antimouse CD80 (B7-1) (16-10A1 clone, Cat No. 553769), fluorescein isothiocyanate (FITC)-rat antimouse CD86 (B7-2) (GL1 clone, Cat. No. 553691), PE-mouse antimouse H-2K^d^ (MHC-I) (SF1-1.1 clone, Cat. No. 553566) and FITC-mouse antimouse I-A^d^ (MHC-II) (AMS-32.1 clone, Cat No. 553547) were purchased from Becton Dickinson-Pharmingen (NJ, USA). FITC-anti-CD3 and PE-anti-CD8 were purchased from Becton Dickinson-Pharmingen. To prepare the CT plant extract used in our experiments, the flowers of the plants were collected and dried. Dried flowers (100 g) were boiled in 1 l distilled water (1 : 10 ratio, w/v) for 1 h and the extract was filtered through Whatman No. 1 filter paper (Whatman, USA). The filtrate was lyophilized into powder and stored at 4°C. The CT powder was dissolved in phosphate-buffered saline (PBS) (pH 7.4) at a stock concentration of 100 mg/mL for *in vitro* experiments.

### 2.2. Cytokine Secretion from Mouse Splenocytes Induced by CT Extract

The splenocytes were homogenized from the spleen of healthy BALB/c mice, and the splenic T lymphocytes were isolated by passage through nylon wool columns [42]. The purity of T cells was confirmed by flow cytometry to be >90%. The mouse splenic T lymphocytes were treated with various concentrations of CT extract (5, 10 and 20 *μ*g/mL of culture medium) for 24 h. The medium was collected to determine the concentrations of the cytokines IL-2, IFN-*γ* and IL-4. Cytokine levels were determined by the DuoSet ELISA kit as described in the manual.

### 2.3. Maturation of Mouse DCs by CT Extract

Bone marrow cells were taken from BALB/c mice and then suspended in RPMI-1640 medium containing 10% FBS. The cell suspension was allowed to stand at room temperature for 10 min to remove the cell clots, and then the cell suspension was centrifuged at 800 g for 5 min at 4°C and washed with PBS (pH 7.4) containing 0.5% FBS and 2 mM EDTA. The cells were allowed to attach to a 100-mm culture plate for 2 h and the floating cells were removed gently. The cells were then treated with GM-CSF (100 ng/mL) and IL-4 (100 ng/mL) at 37°C, 5% CO_2_ for 6 days, following the modifications of Zheng et al. [43]. After 6 days of differentiation, the DCs were treated with various concentrations of CT extract (5, 10 and 20 *μ*g/mL of culture medium) for 72 h. After CT treatment, the DCs were collected for flow cytometric analysis of surface immunological molecules (CD80, CD86, MHC-I and MHC-II). In brief, the cells were stained with the specific FITC-conjugated antibody.

### 2.4. Isolation of CD117^+^ Bone Marrow Cells

Bone marrow cells were collected as above and suspended in PBS (pH 7.4) containing 0.5% FBS and 2 mM EDTA prior to the isolation of CD117^+^ cells. CD117^+^ cells were isolated using magnetic-activated cell sorting (Miltenyi Biotec Inc, CA, USA) as described in the manual. Briefly, 1 × 10^7^ cells were added to 20 *μ*l anti-CD117^+^ provided by the kit for 15 min and then washed with PBS to remove excess unbound antibodies. The treated cells were loaded on a column, from which the CD117^−^ cells were eluted with PBS buffer in the presence of a magnetic field. The CD117^+^ cells were then eluted in the absence of a magnetic field and were then differentiated to DCs by adding GM-CSF and IL-4 as described above.

### 2.5. Generation of CD117^+^-Derived DCs

The CD117^+^ cells were cultured at a density of 6.25 × 10^7^/well of a 6-well plate in RPMI-1640 medium containing 10% FBS, 1.5 mg/mL sodium bicarbonate, 0.1 *μ*mol/mL nonessential amino acids, 1 *μ*mol/mL sodium pyruvate, 100 U/mL penicillin G and 100 *μ*g/mL streptomycin supplemented with GM-CSF (100 ng/mL) and IL-4 (100 ng/mL) at 37°C, 5% CO_2_ for 6 days. Medium containing GM-CSF and IL-4 was refreshed every 2 days.

### 2.6. Assessment of Cytokines Released from JC Lysate-Pulsed CT-Treated CD117^+^-Derived DCs

After differentiation of CD117^+^ cells to DCs for 6 days, DCs were pulsed with JC-lysate and treated with or without CT extract (5, 10 and 20 *μ*g/mL) for an additional 3 days. The conditioned medium was collected for determination of the concentrations of IL-2, IL-10, TNF-*α* and IL-1*β* [44].

### 2.7. Assessment of Immunological Molecules of JC Lysate-Pulsed CT-Treated CD117^+^-Derived DCs

After differentiation of CD117^+^ cells to DCs for 6 days, DCs were pulsed with JC lysate and treated with or without CT extract (20 *μ*g/mL) for an additional 72 h. After CT treatment, DCs cell were analyzed for cell surface markers (CD80, CD86, MHC-I and anti-MHC-II) by flow cytometry.

### 2.8. Preparation of DC Vaccine and JC Tumor Animal Experiment

CD117^+^ cells were cultured in differentiation medium (GM-CSF and IL-4) for 6 days followed by cultivation in fresh RPMI medium in the presence or absence of CT (20 *μ*g/mL) for 3 days. JC cells (3-4 × 10^6^) were lyzed in 1 mL RPMI-1640 medium containing 10% FBS by freezing and thawing four times, and the lysate was stored at –80°C as a tumor antigen resource [45] to pulse the DCs.

BALB/c female mice (4–6 weeks) were purchased from the National Laboratory Animal Center (Taiwan, ROC). JC cells (3 × 10^6^ per 150 *μ*l PBS) were subcutaneously inoculated in the flank of BALB/c mice. Twenty-four tumor-bearing mice were grouped (*n* = 8/group) as follows: untreated control, DC vaccine and CT-treated groups. In the DC vaccine group and CT-treated DC vaccine group, mice were injected intraperitoneally with the JC-pulsed DC cell suspension (1.7 × 10^6^ per 0.2 mL PBS) and the CT-treated JC-pulsed DC cell suspension (1.7 × 10^6^ per 0.2 mL PBS) on day 13, respectively. The tumor growth of JC tumor-bearing mice was measured by a caliper every 4–6 days and calculated using the equation, tumor weight (mg) = length (mm) × width (mm^2^)/2, until the tumor reached 2 × 10^3^ mm^3^. On day 32, mice were euthanized by CO_2_, the tumors were excised and weighed and the spleens of each mouse were homogenized into a single cell suspension pulsed with or without CT-treated DCs to measure Th1- and Th2-related cytokines. The splenocytes were also re-stimulated with the previous corresponding JC-pulsed DC vaccine and CT-treated JC-pulsed DC vaccine for flow cytometric analysis of cytotoxic T lymphocytes. All mice received humane care, and the study protocol followed the guidelines of the Institutional Animal Care and Use Committees of the Development Center for Biotechnology (accredited by AAALAC).

### 2.9. Effects of CT-Treated DCs on the Cytokine Secretion and Cytotoxic T Lymphocytes of Mouse Splenocytes

DC vaccine was prepared as described above and cultured in AIM-V medium (Gibco, USA), and splenic T lymphocytes were taken from the normal and the JC tumor-bearing mice and cultured in RPMI-1640 medium (Gibco, USA). In brief, splenic T lymphocytes were partially purified using a nylon wool column [46]. Different cytokines in response to CT (20 *μ*g/mL)-treated DCs were determined when DCs (2 × 10^5^/mL) were pulsed with splenocytes from tumor-bearing mice at a cell : cell ratio of 1 : 10, 1 : 20 and 1 : 40 for 48 h at 37°C, 5% CO_2_. After cocultivation of splenocytes and DCs, the medium was collected and stored at –20°C prior to cytokine analysis. Cytokines were analyzed by ELISA as described above. The splenocyte culture was continued for an additional 72 h and then subjected to flow cytometric analysis, in which specific T lymphocytes were gated by staining with FITC-anti-CD3 and PE-anti-CD8.

### 2.10. Statistical Analysis

Data were analyzed by one-way ANOVA using SPSS statistical software followed by the Dunnett's test for multiple comparisons between each group. A *P*-value < .05 was considered to indicate a significant difference.

## 3. Results

### 3.1. IFN-*γ* and IL-10 Secretion from Mouse Splenic T Lymphocytes

**Figure 1:**
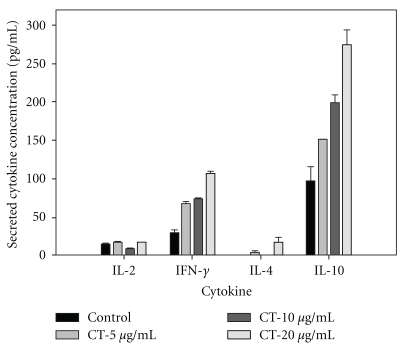
CT extract stimulates cytokine secretion of mouse splenic T lymphocytes. Splenocytes were homogenized from the spleen of healthy BALB/c mice. Mouse splenic T lymphocytes were treated with various concentrations of CT extract for 24 h, and then the medium was assessed for secretion of IL-2, IFN-*γ*, IL-4 and IL-10, as determined by ELISA. The assay was performed in triplicate, and the data are expressed as mean ± SE.

To determine the effect of CT extract on the stimulation of mouse resting T lymphocytes, the splenic T lymphocytes were partially purified and treated with various concentrations of CT extract for 24 h. The supernatants were collected and evaluated for the levels of IL-2, IFN-*γ* and IL-4 and IL-10.  Figure 1 shows that CT extract stimulated the production of IL-10 > IFN-*γ* in a dose-dependent manner (Figure 1). However, no such stimulation was observed for IL-2 and IL-4.

### 3.2. Expression of Immunological Molecules in the Immature DCs

**Figure 2:**
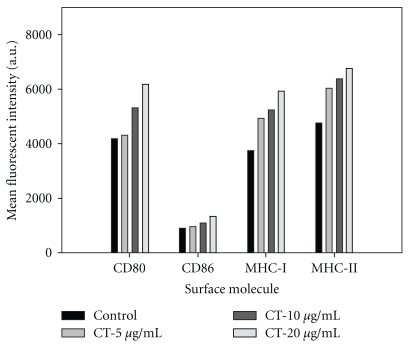
CT extract enhances the maturation of mouse DCs. Mouse bone marrow cells were collected as described in Section 2. After 6 days of differentiation with GM-CSF and IL-4, the cells were treated with various concentrations of CT extract in the absence of GM-CSF and IL-4 for an additional 48 h. The cells were then scraped and collected for surface molecule analysis (CD80, CD86, MHC-I and MHC-II) using flow cytometry.

To determine whether the CT extract stimulated the maturation of DCs, mouse bone marrow cells were isolated and differentiated into immature DCs by adding GM-CSF and IL-4 for 6 days. The immature DCs were treated with various concentrations of CT extract (5, 10 and 20 *μ*g/mL) for 48 h.  Figure 2 shows that CT extract promoted the expression of DC surface markers (CD86 < MHC-I = MHC-II = CD80) in a dose-dependent manner (Figure 2).

### 3.3. Enhancement of Maturation of DCs by CT Extract

**Figure 3:**
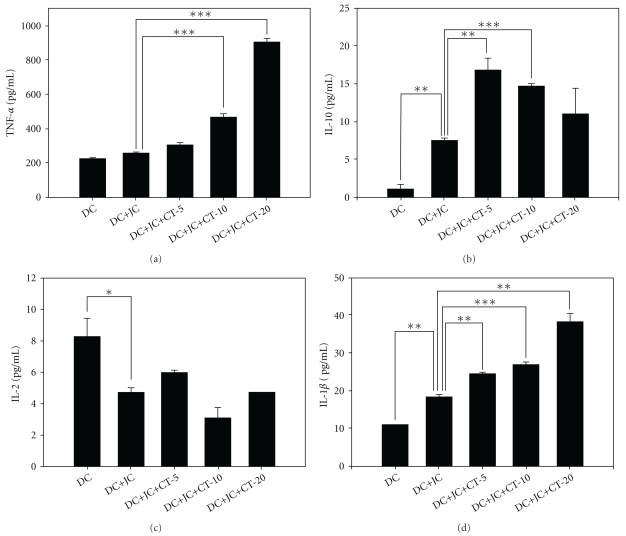
CT extract enhances the maturation of CD117^+^-derived DCs in the presence of tumor antigen. Mouse bone marrow cells were collected as described in Section 2. After 6 days of differentiation with GM-CSF and IL-4, the cells were pulsed with JC tumor lysate (1.7 × 10^6^ per 0.2 mL PBS; DC + JC) and treated with various concentrations of CT extract (5, 10, and 20 *μ*g/mL; DC + JC + CT-5, DC + JC + CT-10 and DC + JC + CT-20) in the absence of GM-CSF and IL-4 for additional 48 h. The medium was collected, and the concentrations of secreted cytokines were determined. Data from triplicate points are expressed as the mean ± SE. Statistical analysis was performed by one-way ANOVA followed by Dunnett's test. A *P*-value < .05 was considered to reflect a significant difference. *P < .05 , **P < .01, ***P < .001.

**Figure 4:**
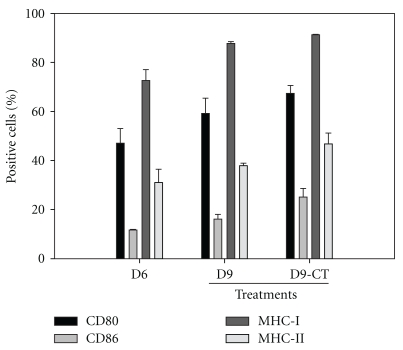
CT extract enhances the maturation of CD117^+^-derived DCs. CD117^+^ cells were isolated as described in Section 2 and then cultured in differentiation medium (GM-CSF and IL-4). After 6 days of differentiation, the differentiated cells (D6) were pulsed with JC tumor lysate (1.7 × 10^6^ per 0.2 mL PBS) in the absence of differentiation cytokines for additional 72 h (D9) or pulsed with JC tumor lysate and incubated with 20 *μ*g/mL CT for additional 72 h (D9-CT). The CD117^+^-derived DCs were then collected and analyzed by flow cytometry for the expression of surface immunological molecules. Experiments were performed in triplicate, and the data are expressed as mean ± SE.

To study whether the pattern of cytokine secretion was altered in DCs stimulated by CT extract in the presence of tumor antigens, the immature DCs were pulsed with JC tumor lysate along with different concentrations of CT extract (5, 10 and 20 *μ*g/mL) for 48 h. The conditioned medium was analyzed for IL-2, IL-10, TNF-*α* and IL-1*β* by ELISA and the DCs were subjected to analysis of surface immunological molecules (CD80, CD86, MHC-I and MHC-II). The results showed that CT extract increased TNF-*α* and IL-1*β* levels in a dose-dependent manner (Figure 3). However, the level of IL-2 was reduced when DCs were pulsed with tumor lysate. IL-10 decreased with an increase in the concentration of CT extract, suggesting that CT extract might increase the secretion of TNF-*α* and IL-1*β* to enhance the maturation of immature DCs. Treatment with CT extract maintained the high profile of cell surface markers, such as CD80, CD86, MHC-I and MHC-II on the CD117^+^-derived CDs in the presence of tumor antigen (Figure 4).

### 3.4. Inhibition of Tumor Growth by CT Treated-DC Vaccine

**Figure 5:**
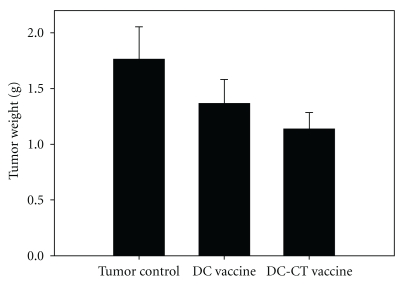
Antitumor activity of the CT extract-treated DC vaccine in JC tumor-bearing mice. DC vaccine or CT-treated DC vaccine (DC-CT) was administered via intraperitoneal injection on day 13 when mean estimated tumor weight was >63 mg (0.5 × 0.5 cm in diameter). A group of tumor-bearing animals without treatment was the tumor control. Animals were sacrificed on day 32, and the tumors were excised and weighed. Data are expressed as mean ± SE (*n* = 8 in each group).

To study the antitumor activity of CT-treated DC vaccine, BALB/c mice were inoculated with JC tumor cells and challenged with a single dose of CT-treated DC vaccine. The JC tumor-bearing mice were challenged with DC vaccine and CT-treated DC vaccine on day 13, when tumor reached the size of 5 mm (length) × 5 mm (width). Results showed that CT-treated DC vaccine and DC vaccine decreased the tumor mass by 32.5% and 17.2%, respectively, compared to the untreated control (Figure 5). This result suggested that CT might increase the antitumor activity of the DC vaccine.

### 3.5. Polarization of Cytokine Secretion to Th1 Response

**Figure 6:**
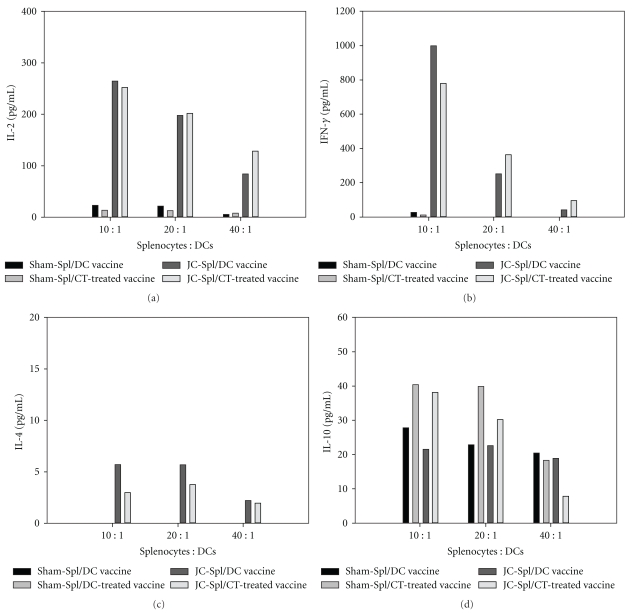
Secretion of cytokines from mouse splenocytes re-stimulated with CT extract-treated DC vaccine *ex vivo*. Spleens from sham (sham-spl) and tumor-bearing (JC-spl) BALB/c mice challenged with DC vaccine or CT-treated DC vaccine were taken and homogenized into single cell suspensions. The splenocytes were cocultured with DC vaccine or CT-treated DC at cell : cell ratios of 10 : 1, 20 : 1 and 40 : 1 for 48 h. The concentrations of secreted cytokines were determined by ELISA as described in Section 2.

To determine the Th1 and Th2 cytokine secretion in DC vaccine treatment, the tumor-bearing mice that had been challenged with DC vaccine were sacrificed and their splenocytes subjected to cytokine analysis, in which those cells were re-stimulated with the previous corresponding DC vaccine or CT-treated DC vaccine *ex vivo*. Splenocytes of the CT/DC vaccine compared with those of the CT-untreated DC-vaccine showed increased or identical production of IL-2, IL-10 and IFN-*γ*. However, IL-4 production was notably decreased in splenocytes of mice treated with the CT/DC vaccine (Figure 6). The secretion of cytokines was not increased in the sham splenocytes when those cells pulsed with DC vaccine or CT-treated DC vaccine with lack of JC antigen. This result suggested that either the DC vaccine or CT-treated DC vaccine elicited antitumor activity through a Th1 response, and that CT extract might also enhance the immune response of the DC vaccine.

### 3.6. Increased Antitumor Activity Is Mediated by Cytotoxic T Lymphocytes

**Figure 7:**
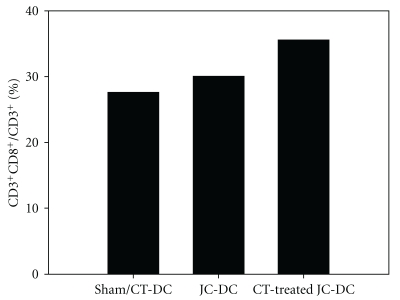
*Ex vivo* stimulation of specific T lymphocytes. The spleens of sham and tumor-bearing mice were taken and homogenized into single-cell suspensions. The splenocytes were re-stimulated with their corresponding treatments (DC vaccine or CT-treated DC vaccine) for 5 days. The splenocytes were then subjected to flow cytometric analysis, and specific T lymphocytes were gated by staining with FITC-anti-CD3 and PE-anti-CD8.

To determine whether cytotoxic T lymphocytes were increased in the CT-treated DC vaccine, the splenocytes of vaccine-challenged mice were re-stimulated with the previous corresponding DC vaccine or CT-treated DC vaccine. The JC tumor lysate-pulsed DC vaccine stimulated 30.1% of cytotoxic T lymphocytes and the JC tumor lysate-pulsed CT-treated DC vaccine stimulated 35.6% of cytotoxic T lymphocytes (Figure 7), indicating that CT-treated JC tumor lysate-pulsed DC vaccine could increase more cytotoxic T lymphocytes against tumors than DC vaccine without CT treatment. Additionally, the cytotoxic T lymphocytes of sham mice were only stimulated 27.6% with CT-treated DC vaccine in the absence of JC tumor antigens. This result was consistent with the decrease in tumor weight shown in Figure 5.

## 4. Discussion

DCs are professional antigen-presenting cells that have been enlisted for use in vaccines against cancer. DCs can acquire tumor antigens by cocultivation with tumor cells or pulsation with tumor lysates to become activated. After maturation, the DCs present the tumor antigens to lymphocytes and trigger the immune response cascade. In this study, CT stimulated splenic T lymphocytes to secrete IFN-*γ* and IL-10, suggesting that CT can immunomodulate T lymphocyte function. Heightened expressions of CD80, CD86, MHC-I and MHC-II imply that CT stimulates maturation of antigen-presenting cells; moreover, an increase in the expression of MHC-I and II molecules, together with an increase in CD8-positive T cells, suggests that HLA-mediated presentation of tumor antigens accelerates after treatment with CT extract. CT also improved the antitumor activity of the DC vaccine as evidenced by a reduction in tumor weight. The *ex vivo* analysis of cytokine secretion and lymphocyte population suggested that CT polarizes the immune response toward the Th1 pathway by increasing the secretions of IL-2 and IFN-*γ*, but not IL-4, and consequently produces more cytotoxic T lymphocytes to elicit antitumor activity than DC vaccine without CT treatment. It has been reported that stimulated DCs secrete TNF-*α*, IL-1*β* and IL-10 during the maturation process and then polarize the T lymphocytes toward the Th1 pathway [47], which is consistent with our observations shown in Figure 3.

DCs are widely distributed in the human body with different morphologies, such as Kupffer cells in liver [48] and Langerhans cells in the skin [49]. Unlike chemotherapy which produces severe side effects during treatment, the use of DCs provides an alternative strategy against tumors [50]. The functions of DCs are improved in such as the maturation, antigen presentation and regulatory cytokine secretion, which has survival benefits in cancer patients [51]. Although the CT-treated DC vaccine was intraperitoneally injected to activate immunity in this study, oral administration of CT extract might promote the recognition of antigens and facilitate antigen presentation via intestinal DCs, and thus this attractive approach is worthy of further investigation. However, many traditional medicinal herbals have being studied for immunomodulatory activities, such as *Melilotus suaveolens Ledeb* [52] and *Tanacetum parthenium* [53]. In literatures, both herbal plants were found to demonstrate their anti-inflammation activities in a monocytic cell-based assay system and exhibited effects on regulating the production of chemokines. Due to these recent studies, *M. suaveolens Ledeb* [52] and *T. parthenium* have been implicated to exhibit potential therapeutic benefits in treating many inflammation-related diseases, such as cancers, atherosclerosis and rheumatoid arthritis. Therefore, herbals in treating cancers might have activities via the modulation of cytokine profiles. In this article, our results suggest that CT could promote immunity through the activation of DCs *per se* that do not alter the cytokine secretion during immune responses of tumor lysate-pulsed DCs toward the Th1 pathway.

## Figures and Tables

**Table 1:** The biological activities and therapeutic indications for extracts of CT.

Plant part	Possible active ingredients	Biological activities and clinical relevance	Literature source
Flower			
	Safflor yellow	Inhibitory effects on the production of antibody and delayed-type hypersensitivity reaction *in vivo*; and the responsiveness of mixed lymphocyte reaction and production of IL2 *in vitro*	Lu et al. [7]
		Antilipid peroxidation effects	Jin et al. [8]
	Triterpene	Anti-inflammatory activity against 12-*O*-tetradecanoylphorbol-13-acetate-induced inflammation	Akihisa et al. [9]
	Polysaccharides	Antitumor activities through toll-like receptor 4/NF-*κ*B pathway in the cell assay	Ando et al. [10]
	Hydroxysafflor yellow A	Anti-thrombotic and -infraction effects and as a neuroprotective agent against cerebral ischemic damage	Wang et al. [1] Zhu et al. [2] Zhang et al. [11] Zhu et al. [12, 13]
	Carthamin, safflower yellow	Antiproliferative and pro-apoptotic activities in hepatic stellate cells	Chor et al. [14]
		Reduction of cerebral infarction volume along with increase of bcl-2 and decrease of caspase-3	Luo et al. [15]
		Improving functions of cardiac contraction and dilation, increasing coronary blood flow and strengthening the bcl-2 protein expression	Zhang and Jiang [16]
	Water soluble substances	Cytotoxic effects on the rat nervous cells and may cause teratogenesis	Nobakht et al. [17]
		Antimycotic properties especially against *Aspergillus fumigatus*	Blaszczyk et al. [18]
Seed			
	Oil extracts	Antitumor effects in a skin and beast cancer animal model and in a melanoma cell assay system	Loo et al. [3] Roh et al. [4] Yasukawa et al. [5]
	Ethanol-ethyl acetate extract (serotonin derivatives and their glucoside)	Immunomodulatory effects mainly associated with anti-inflammatory activities	Takii et al. [6]
	Serotonin derivatives	Antioxidant effects mainly for prevention of atherosclerosis through inhibition of LDL oxidation and postischemic myocardial dysfunction	Koyama et al. [19] Hotta et al. [20]
	Trechelosides	Anabolic effects and antiestrogenic activities on bone through promotion of osteoblastic differentiation and inhibition of bone resorption	Kim et al. [21] Jang et al. [22] Yoo et al. [23] Yuk et al. [24] Hong et al. [25]
	Linoleate	High linoleate in dietary oil supplements increase serum concentrations of prostaglandin F2*α* metabolite	Grant et al. [26]
Leaf			
	Flavonoids	Antioxidative effects	Lee et al. [27]
	Kinobeon A	A preventive effect on singlet oxygen and acting as a tyrosinase inhibitor	Kambayashi et al. [28] Kanehira et al. [29, 30]
